# Supplementary material for: The effect of intrapartum prolonged oxygen exposure on fetal metabolic status: secondary analysis from a randomized controlled trial
Source: Front Endocrinol (Lausanne). 2023 Jun 27;14:1204956. doi: 10.3389/fendo.2023.1204956 (PMC10335765; doi:10.3389/fendo.2023.1204956)
Supplement: Supplementary file 3 [file DataSheet_3.zip › Result-X101SC21092977-Z01-J001-B1-42/4.MetDiffAnalysis/O.vs.A/O.vs.A_neg_cluster_heatmap.pdf]

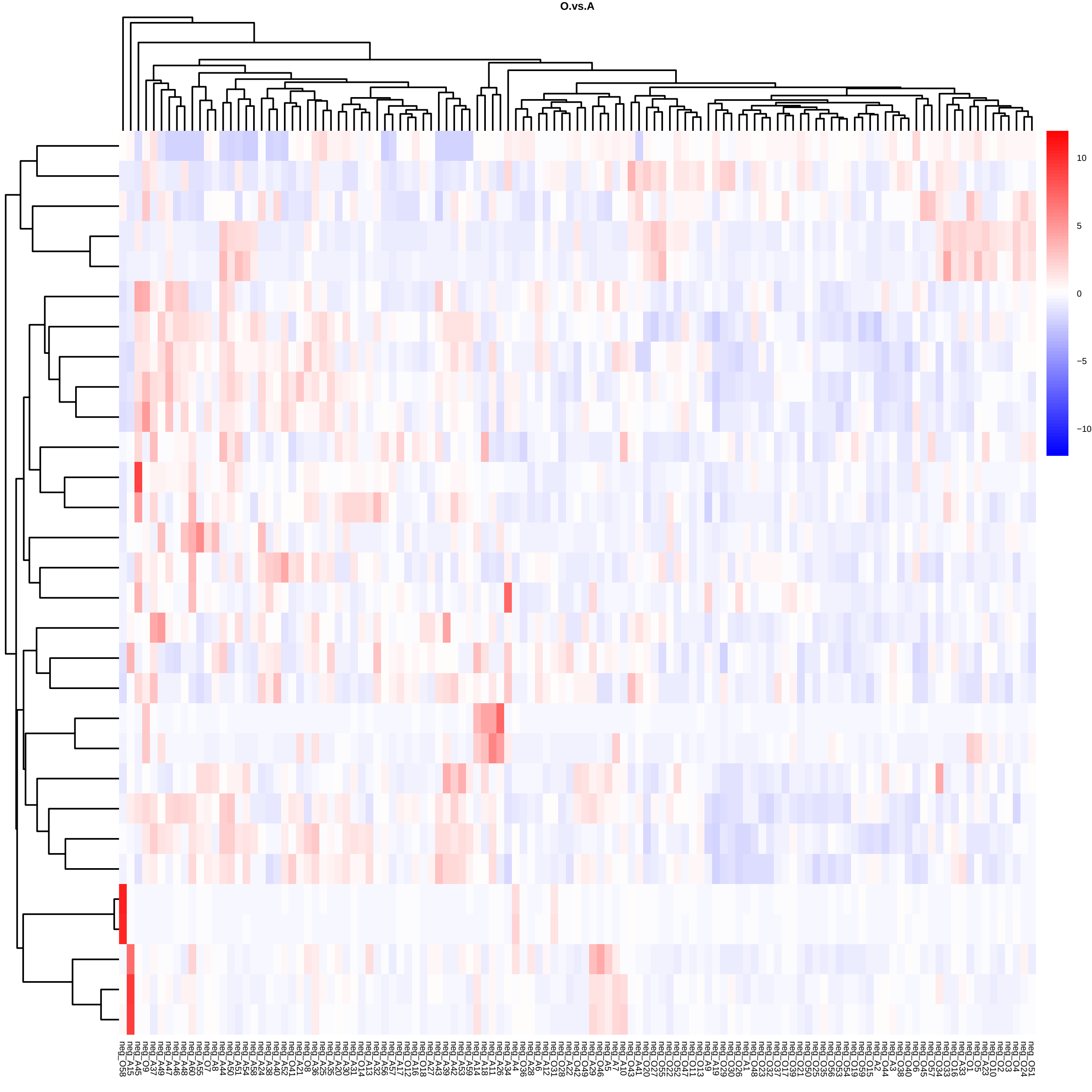

O.vs.A
10
5
0
−5
−10
neg_O58
neg_A15
neg_A45
neg_O9
neg_A37
neg_A49
neg_A47
neg_A46
neg_A48
neg_A60
neg_A55
neg_O7
neg_A8
neg_A44
neg_A50
neg_A51
neg_A54
neg_A58
neg_A24
neg_A38
neg_A40
neg_A52
neg_O41
neg_A21
neg_O8
neg_A36
neg_A25
neg_A35
neg_A20
neg_A30
neg_A31
neg_O14
neg_A13
neg_A32
neg_A56
neg_A57
neg_A17
neg_O12
neg_A16
neg_O18
neg_A27
neg_A43
neg_A39
neg_A42
neg_A53
neg_A59
neg_A14
neg_A18
neg_A11
neg_A26
neg_A34
neg_A4
neg_O36
neg_A28
neg_A6
neg_A12
neg_O31
neg_O28
neg_A22
neg_O42
neg_O49
neg_A29
neg_O46
neg_A5
neg_A7
neg_A10
neg_O43
neg_A41
neg_O20
neg_O27
neg_O55
neg_O22
neg_O52
neg_O47
neg_O11
neg_O13
neg_A9
neg_A19
neg_O29
neg_O30
neg_O26
neg_A1
neg_O48
neg_O23
neg_O32
neg_O37
neg_O17
neg_O39
neg_O21
neg_O50
neg_O25
neg_O35
neg_O56
neg_O53
neg_O54
neg_O19
neg_O59
neg_O15
neg_A2
neg_O44
neg_A3
neg_O38
neg_O40
neg_O6
neg_O45
neg_O57
neg_O34
neg_O33
neg_O16
neg_A33
neg_O1
neg_O5
neg_A23
neg_O10
neg_O2
neg_O3
neg_O4
neg_O24
neg_O51
